# R Package for Calculating Estimators of the Proportion of Explained Variance and Standardized Regression Coefficients in Multiply Imputed Datasets

**DOI:** 10.1177/01466216251316275

**Published:** 2025-01-26

**Authors:** Joost R. van Ginkel, Julian D. Karch

**Affiliations:** 1 100575 Leiden University, Leiden, The Netherlands

**Keywords:** missing data, multiple imputation, regression, proportion of explained variance, standardized regression coefficients

## Description

Multiple imputation (Rubin, 1987) has become a widely recommended way to deal with missing data. The procedure consists of the following three steps: (1) the missing data are estimated multiple times, which results in multiple plausible complete versions of the incomplete dataset, (2) the completed datasets are analyzed using the intended statistical analysis, and (3) the results of these several analyses are combined into a pooled analysis using specific combination rules. Until recently, no combination rules were proposed for standardized regression coefficients and the proportion of explained variance, *R*^2^, in multiple regression analysis. However, Van Ginkel (2020) proposed combination rules for both these statistics, along with combination rules for confidence intervals for standardized regression coefficients. Additionally, Van Ginkel and Karch (2024) extended the combination rules for *R*^2^ by Van Ginkel (2020) to 10 alternative estimators for the proportion of explained variance, among which the Ezekiel estimator, better known as *R*^2^-adjusted, the Smith estimator, and the Wherry estimator.

To calculate the above statistics for multiply imputed datasets, we developed an R (R Core Team, 2024) package in which the combination rules for these statistics are included. By default, the procedure displays the pooled estimator for *R*^2^ by Van Ginkel (2020), the pooled multiple correlation (the square root of *R*^2^), *R*^2^-adjusted, and the pooled standardized regression coefficients by Van Ginkel (2020). Optionally, the procedure also displays confidence intervals of the pooled standardized regression coefficients, the pooled zero-order correlations of the predictor variables with the outcome variable, and the pooled alternative estimators of the proportion of explained variance.

## Availability

The R package RsquaredMI can be obtained from the Comprehensive R Archive Network (CRAN) repository at https://cran.r-project.org/web/packages/RSquaredMI/. The manual is available at https://cran.r-project.org/web/packages/RSquaredMI/RSquaredMI.pdf. The development version of the package is hosted on GitHub: https://github.com/karchjd/RsquaredMI.
